# The salivary levels of leptin and interleukin-6 as potential inflammatory markers in children obesity

**DOI:** 10.1371/journal.pone.0210288

**Published:** 2019-01-03

**Authors:** Corina Pîrsean, Cătălina Neguț, Raluca-Ioana Stefan-van Staden, Cristina Elena Dinu-Pirvu, Petru Armean, Denisa Ioana Udeanu

**Affiliations:** 1 School of Midwifery and Nursing, University of Medicine and Pharmacy “Carol Davila”, Bucharest, Romania; 2 Laboratory of Electrochemistry and PATLAB Bucharest, National Institute of Research for Electrochemistry and Condensed Matter, Bucharest, Romania; 3 Department of Physical and Colloidal Chemistry, Faculty of Pharmacy, University of Medicine and Pharmacy “Carol Davila”, Bucharest, Romania; 4 Department of Clinical Laboratory. Food Safety, Faculty of Pharmacy, University of Medicine and Pharmacy “Carol Davila”, Bucharest, Romania; Dasman Diabetes Institute, KUWAIT

## Abstract

**Background:**

Obesity among children is an alarming issue due to an increased incidence over the last years with devastating physiological and psychological consequences. Current available medical diagnostic tools use invasive methods to evaluate and monitor the lipid profile, glycaemia or liver status for determining the overweight/ obesity complications. The standard methods proposed for the assay of IL6 and leptin from saliva cannot detect these two biomarkers in children saliva; the levels of IL6 and leptin in children’s saliva are lower than the limit of determination of the standard methods. Therefore, we proposed a method based on utilization of stochastic sensors, able to simultaneously perform a qualitative and quantitative determination of these two biomarkers within minutes, in the range able to cover healthy and obese children.

**Methods:**

Children from the urban area monitored for annual standard analyses and health status assessment at National Institute of Endocrinology C.I. Parhon within University of Medicine and Pharmacy “Carol Davila”, Bucharest, Romania were included in the study. In the same day, for all participants of the study, blood analyses were performed and saliva samples were collected for the determination of the IL-6 and leptin levels.

**Findings/ Results:**

The children diagnosed with overweight/ obesity presented not significantly different blood lipid profile and glycaemia comparing to the control group. Only few cases of the children presented high levels of cholesterol, low level of HDL-cholesterol, a slightly increased level of triglycerides and transaminases. No correlation with the body mass index could be established with the blood analyses results. In case of the overweight/obese children, the salivary level of the proinflammatory citokynes IL-6 (41ng/mL±21) and leptin (40.4ng/mL±28.8), were significantly increased comparing to normal weight children (IL-6 8.1±4.6, leptin 9.58±3.1). Moreover, the saliva level of the IL-6 was positively correlated with the body mass index. Salivary leptin level was highly variable in case of obese children, 6 patients presenting similar levels with the control group.

**Conclusions:**

Increased levels of salivary IL-6 and leptin sustain a systemic inflammation status despite normal range of standard blood analyses. The results were positively correlated in case of IL-6 with the body mass index the general accepted method used for the assessment of the obesity or overweight degree The determination of these markers in saliva samples by a stochastic method proved the utility within the medical examination for a better evaluation of the health status in obesity. The method has some advantages like: easy collection of the biological sample, fast determination of low concentrations and could be promising in case of no associated oral cavity infections or inflammations which could interfere the results.

## Introduction

Obesity is a complex condition with multifactorial etiology, generally considered a disequilibrium of energy intake and expenditure. According to WHO, the prevalence of obesity worldwide is at epidemic dimension, more than 2 billion people suffering of overweight and obesity. Among the adults, the number of obese has doubled since 1980s in both developed and developing countries. An alarming issue is the increased incidence of obesity among children even at very young ages with devastating physiological and psychological consequences. Unfortunately, many parents still ignore the gravity of the overweight and obesity in children, most of them being diagnosed when complications are already present, and the quality of life is affected [1, 4].

The obesity is a known risk factor for developing several comorbidities like diabetes mellitus, cardiovascular diseases, cancer and metabolic syndrome [2,3].

Adipose tissue has an important role in depositing the nutrients excess, sensing the nutrients status and is involved in the regulation of the energy expenditure [1,4,5]. This tissue has a limited capacity of storage, overnutrition generating local oxidative stress and inflammation [2]. Low grade inflammatory response to obesity is mainly maintain by adipocytes and immune cells secreting a variety of pro-inflammatory signals like interleukin-6, adipokines, leptin, resistin, TNF-α, monocyte chemoattractant protein-1 (MCP-1) [3,6]. All this signaling molecules are involved in chronic systemic inflammation, insulin resistance, adipogenesis, atherosclerosis with severe consequences like diabetes, cardiovascular diseases, metabolic dysfunction and risk in development of severe asthma [2,3,7–9].

BMI is the generally accepted method of diagnosing and assessing the obesity degree. Some standard blood analyses are also determined for monitoring the health status and earlier onset of complications. All these routine analyses are useful in diagnosing some comorbidities like diabetes or cardiovascular disease but there is a less prediction in the assessment of the inflammation process which triggers the complications.

Developing new non-invasive methods for analyzing other parameters better correlated with obesity complications than standard parameters are of great interest especially in patients of young ages.

Over the last years, saliva has been studied as an alternative biological fluid suitable for a non-invasive diagnostic. Current medical diagnostic tools use this biological fluid for testing in area of toxicology, infectious disease or endocrinology.

Saliva has a complex chemical composition being generally a mixture of water, electrolytes, different proteins, enzymes and antimicrobial substances provided mainly by salivary glands, upper respiratory mucosa and oral mucosa transudate. Different components of blood have been demonstrated to enter saliva using intracellular mechanisms of passive diffusion or active transport or by ultrafiltration between cell junctions. In case of obese or overweight subjects, several blood biomarkers have already been identified in saliva and their concentrations were correlated with the salivary level like inflammatory cytokines, adipokines, insulin and cortisol [9,10].

Interleukin-6 and leptin are among the biomarkers with strong correlation between the blood and salivary levels [9–12]. Elevated levels of these parameters are frequently encountered in the inflammatory state, metabolic disease and cardiovascular risk associated to obesity [9,10].

Interleukin-6, considered a major inflammatory cytokine secreted in excess by the adipose tissue in case of obesity is involved in signaling pathways of insulin sensitivity, downregulation of lipoprotein lipase, triglycerides synthesis and modulation of the expression of some adipose tissue specific genes [3,13].

Leptin is the key adipokine with mediator role on the adipose tissue-brain signaling pathways involved in obesity etiology, pathophysiology and health outcomes [11,14,15]. The circulating systemic level is proportionally to the amount of adipose tissue and regulates energy homeostasis signaling the energy storage. Inflammation and stress are some of the mechanisms of developing leptin resistance in obese and overweight patients with long term secondary effects. An elevated level of leptin was determined in case of weight gain associated with cardiovascular onset [11,16,17].

These findings sustain the valuable potential of salivary markers determination in developing new diagnostic techniques non-invasive and less stressful especially in case of pediatric patients.

In this context, a new method was previously designed, tested and validated on a preclinical model with promising results in medical practice [18,19]. The method based on stochastic sensors is fast, and reliable and require small volumes of biological samples (up to 50μL of sample). The method allows the determination of low levels of concentration being useful in detecting small concentration of signaling molecules. It is actually the only method working to date for the assay of these types of substance in children’s saliva.

The study aims to determine salivary IL-6 and leptin as obesity associated inflammation markers using a new non-invasive method with clinical applications in case of children diagnosed with overweight and obesity.

## Material and methods

### Reagents and solutions

1-adamantyloleamide used for the design of the stochastic sensor was previously synthesized and described in the literature [20]. Deionized water obtained from a Millipore Direct-Q3 System (Mosheim, France) was used for the preparation of all solutions. Graphite powder (1–2μm) was supplied by Aldrich (Schnelldorf, Germany) and paraffin oil by Fluka (Buchs, Switzerland), monosodium phosphate and disodium phosphate were purchased from Reagent, Bucharest. Working standard IL-6 solutions of 10^−15^ to 10^−6^ g/mL, and leptin solutions of 1.25x10^−14^ to 1.25x10^−5^ g/mL were prepared using the serial dilution method, from standard solutions obtained from Aldrich (Schnelldorf, Germany). When not in use, leptin and IL-6 solutions were stored in the fridge at 2–8°C.

### Apparatus and methods

All measurements were performed using a PGSTAT 302N potentiostat/galvanostat (Metrohm), connected to a classic three-electrode cell, and linked to a computer via an Eco Chemie (Utretch, The Netherlands) software version 4.9. Ag/AgCl (3 mol/L KCl) electrode served as reference electrode and platinum wire electrode served as counter electrode. A stochastic method was used for the measurements of t_*off*_ and t_*on*_, at a constant potential (125 mV vs. Ag/AgCl).

### Study participants

The study included 17 children from urban area, diagnosed with overweight/ obesity and monitored at National Institute of Endocrinology C.I. Parhon within University of Medicine and Pharmacy “Carol Davila”, Bucharest, Romania and 5 children with normal weight who presented to the same institution for annual standard analyses and health status assessment. We excluded from the study, patients diagnosed with other associated disease like endocrine disorders or oral cavity inflammation or infections. All 22 subjects included in the study had the blood analyses performed in the same clinical laboratory and the saliva samples were collected during the same day as blood collection. All analyses were performed in the same day for all participants of the study to avoid the differences between samples regarding the preservation time.

All parents or legal tutors of the children gave their written consent for the saliva samples taken from their children and the study was approved by Ethical Commission of the University of Medicine and Pharmacy “Carol Davila”, Bucharest (Approval no 158/11.03.2018, code PO-35-F-03).

The body mass index (BMI) and percentile was calculated according to the general formula compared with published growth charts [21]. The body weight categories were defined as it follows: obese >95^th^ percentile, overweight 85^th^ -95^th^ percentile and normal weight 5^th^-85^th^ percentile.

### Saliva samples

22 saliva samples (5 controls and 17 samples collected from children diagnosed with overweight/obesity) were collected in the same morning between 8–9 am. All subjects were instructed not to eat, drink or wash the mouth since the evening before collection. Saliva sampling was performed following a mouth rinse with 5 ml of water to wash out any debris or exfoliated cells. From each subject around 2-3ml of unstimulated whole saliva was collected on recipients kept on ice. The samples were centrifuged and aliquots of 1mL were stored at -80°C. The saliva samples were used for the assay of IL-6 and leptin by stochastic sensing without any pretreatment.

### Blood analyses

Same day blood analyses results were provided by the institute and the following biochemical analyses were considered for the study: total cholesterol, HDL-cholesterol, triglycerides, glycaemia and transaminases.

### Statistical analyses

**Fig 1:**
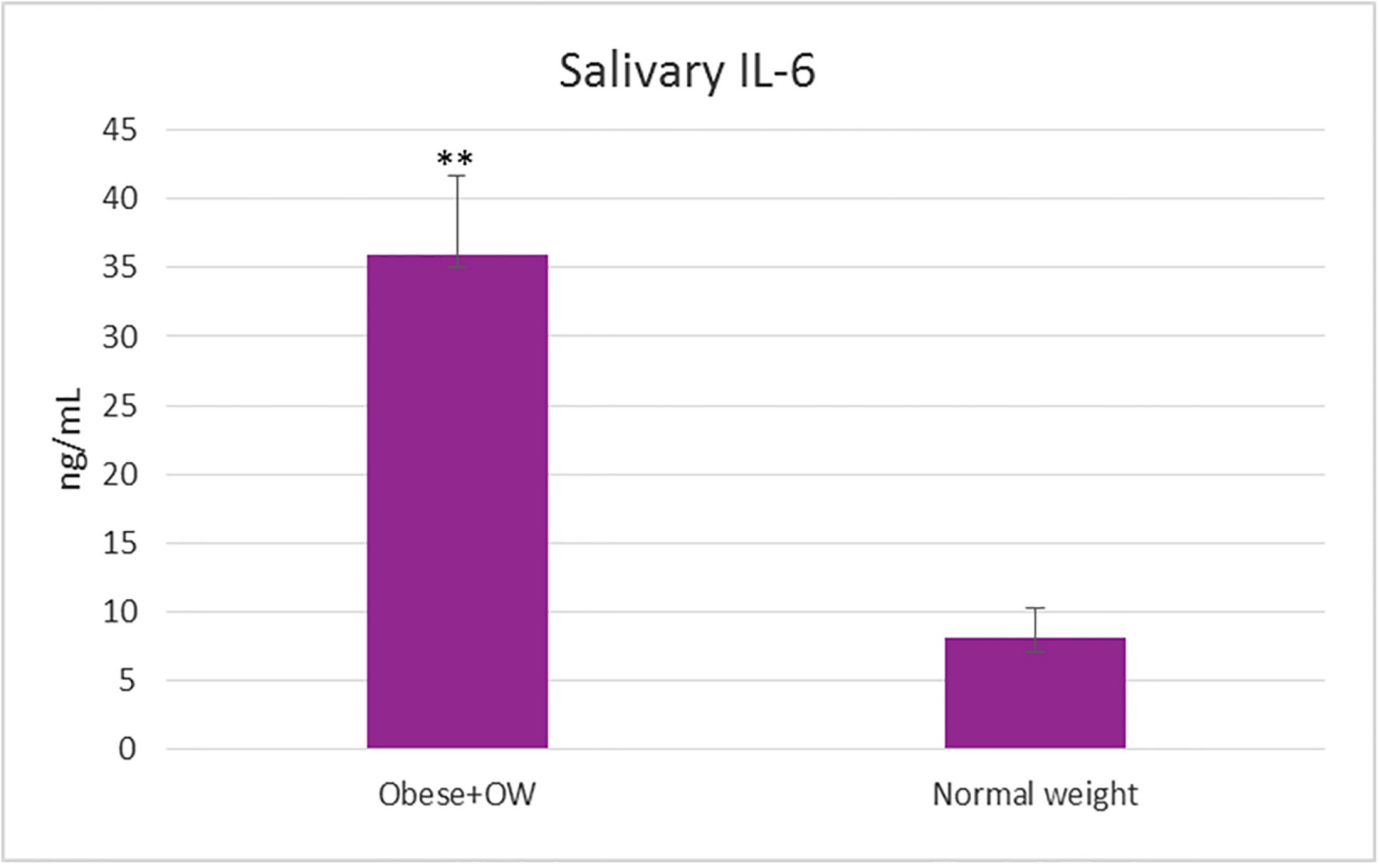
The level of the salivary IL-6 determined by the new stochastic sensing method in case of obese/overweight children and control group with normal weight. (**t-test statistical significance p<0.01 vs. control).

**Fig 2:**
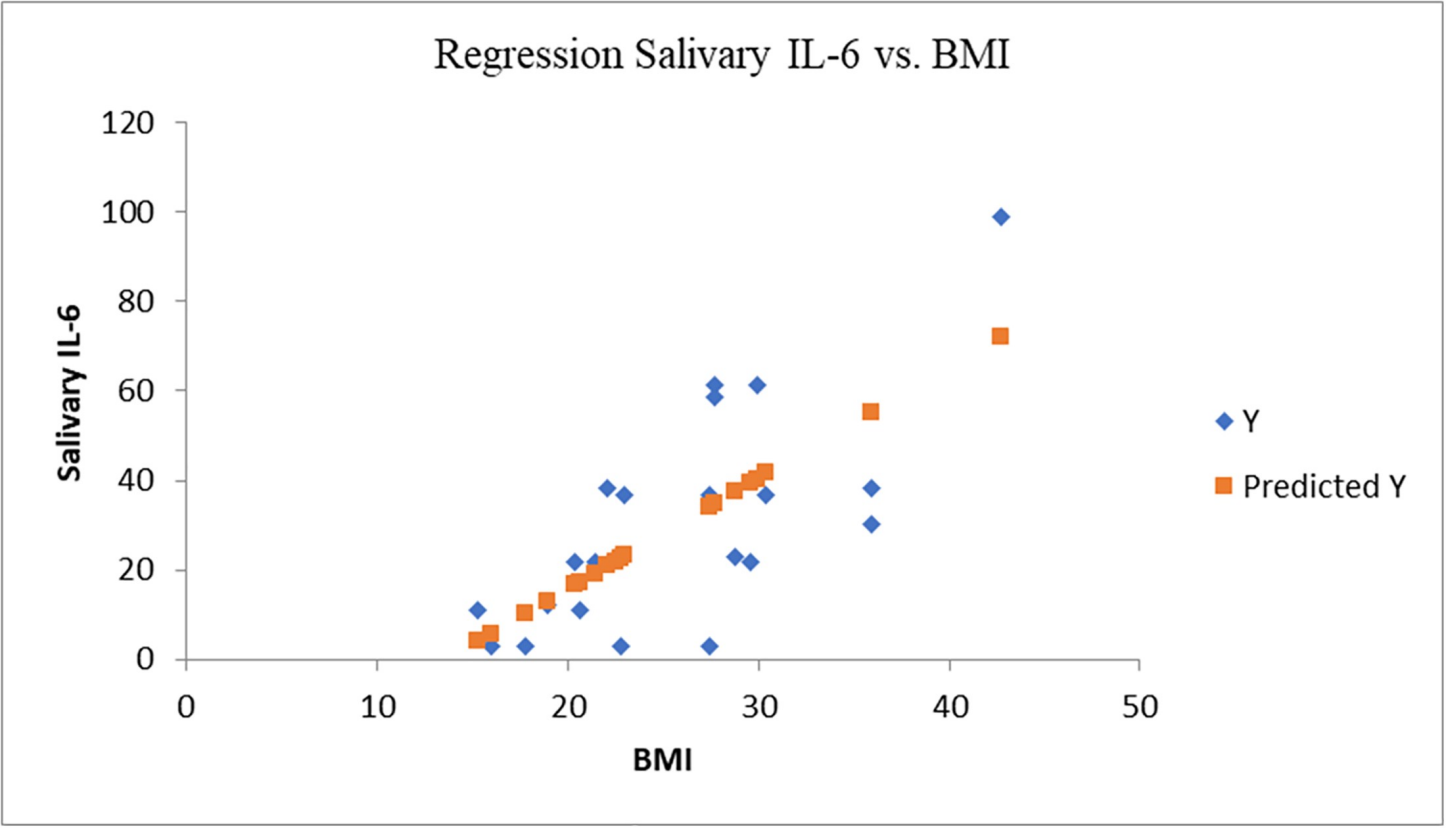
Statistical regression IL-6 vs. BMI (multiple r = 0.71, r square = 0.51).

**Fig 3:**
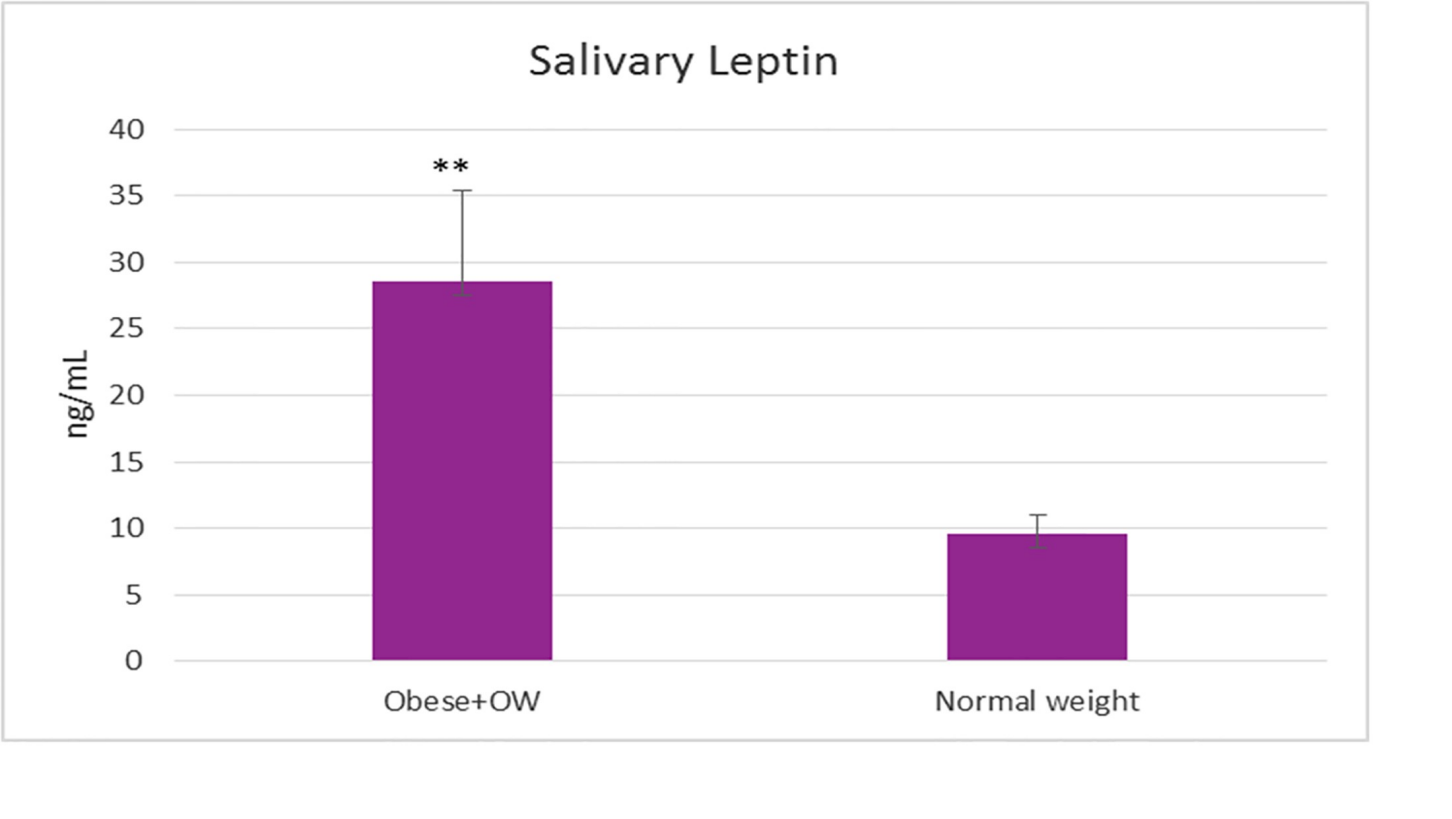
The level of salivary leptin determined by the new stochastic sensing method in case of obese/overweight children and control group with normal weight. (**t-test statistical significance p<0.01 vs. control).

**Fig 4:**
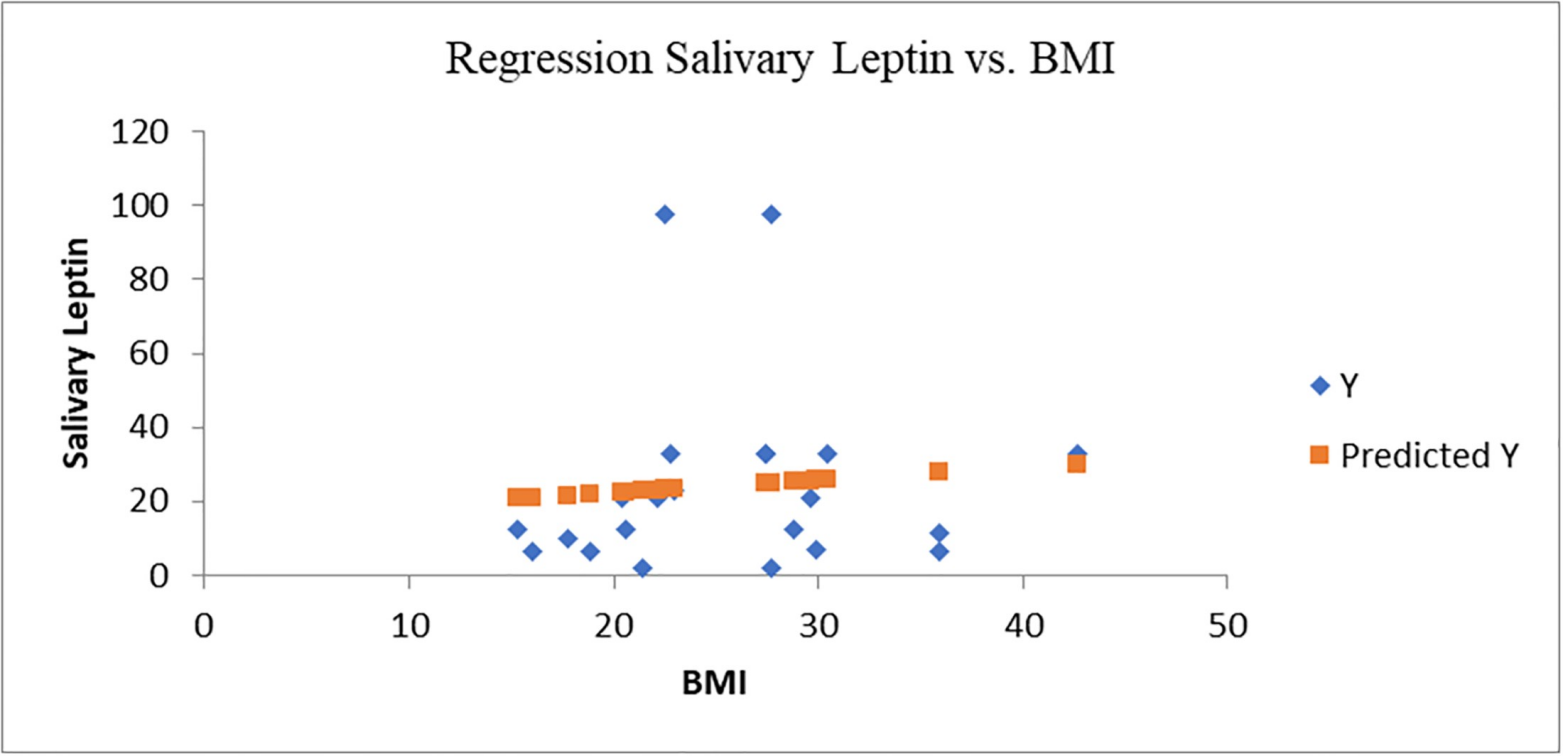
Statistical regression salivary leptin vs. BMI (multiple r = 0.09, r square = 0.008).

**Fig 5:**
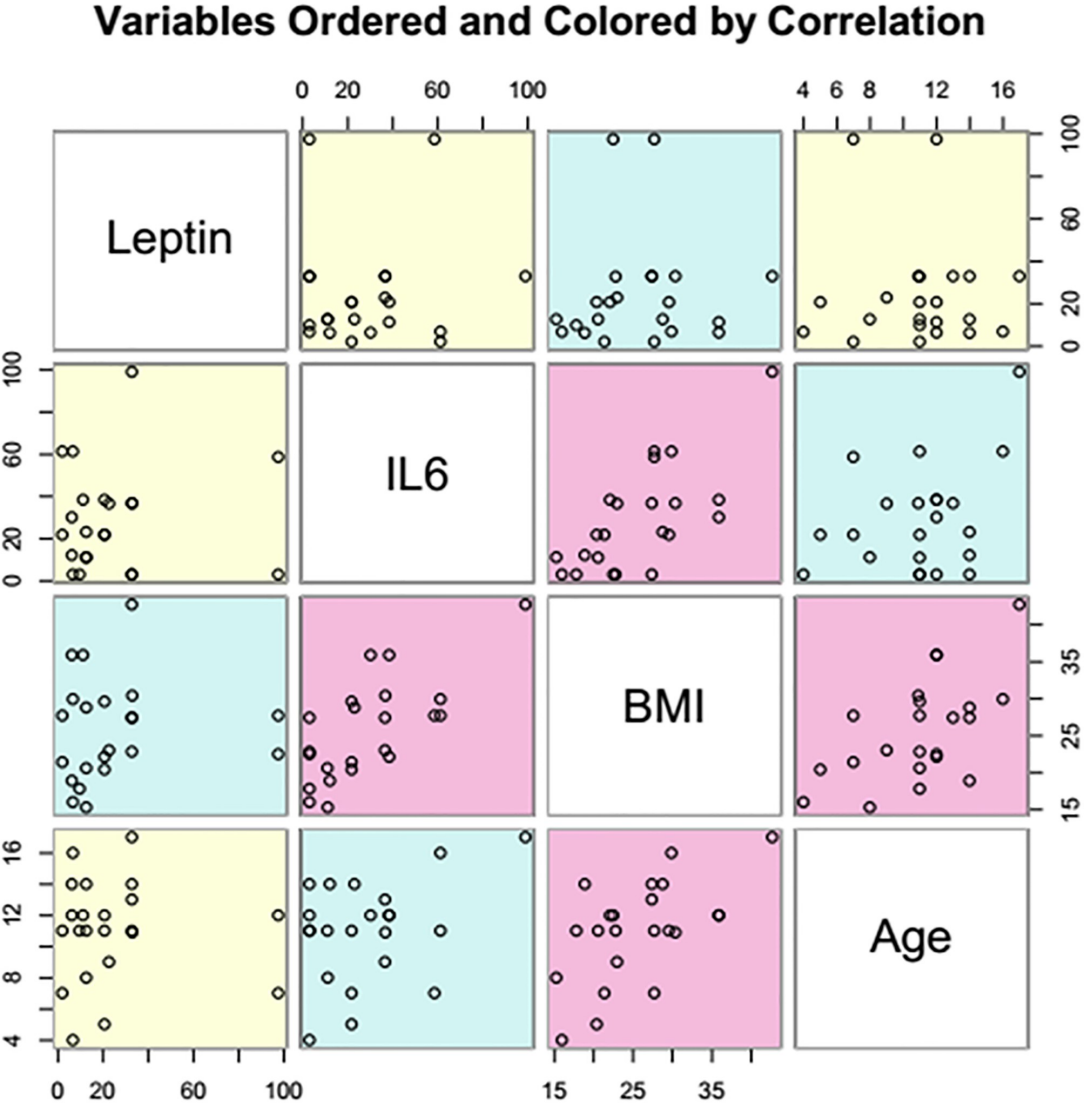
Correlation of the variables: Leptin, IL-6, BMI and age in case of children selected for the study (pink—Strong correlation, blue—Weak correlation, yellow—No correlation).

Statistical analyses for all figures (Figs 1–5), and Table 2 were performed using two softwares GraphPad Prism version 6 for Windows (La Jolla California USA) and R Project for statistical computing version 3.5.1. for Windows. GraphPad Prism was used to calculate common statistic parameters like standard deviation, standard error and statistical significance using unpaired t-test. Data are presented as mean±SD, unless otherwise is indicated. For all analyses, a P value<0.05 is considered statistical significant. Multiple regression and correlation analyses were performed using R simulation programme to determine the association between different variables. The value of correlation coefficient *r* ranges from -1 to 1, value 0 meaning there is no linear correlation between the variables.

## Results

Obesity degree is classified using the body mass index which in case of children, calculated BMI is compared with growth charts. The body mass index, age, weight, height and sex of the children diagnosed with obesity/ overweight and healthy weight included in the study are presented in Table 1.

**Table 1:** Body mass index (BMI), age, weight, height and sex of the children diagnosed with obesity/ overweight and healthy weight included in the study.

Child	Diagnostic	BMI	Percentile	Age	Weight	Height	Sex
**1**.	Obese	42.7	>99th	17	109.6	160.2	F
**2**.	Obese	35.9	99th	12	85.7	154.4	F
**3**.	Obese	35.9	>99th	12	85.7	154.4	M
**4**.	Obese	30.4	>99th	10.9	76.4	158.6	M
**5**.	Obese	29.9	96th	16	75.7	159	F
**6**.	Obese	29.6	98th	11	70	153.7	M
**7**.	Obese	28.8	97th	14	77	163.5	M
**8**.	Obese	27.7	>99th	7.7	44	126	M
**9**.	Obese	27.7	98th	11	71	160	M
**10**.	Obese	27.4	96th	14	56.6	143.6	F
**11**.	Obese	27.4	97th	13	76.5	167	M
**12**.	Obese	23	96th	9	43.1	137	F
**13**.	OW	22.8	94th	11	59	161	M
**14**.	OW	22.5	88th	12	48	146	F
**15**.	OW	22.1	90th	12	58	162	M
**16**.	Obese	21.4	97th	7	35	128	F
**17**.	Obese	20.4	>99th	5	26	113	M
**18**.	HW	20.6	83rd	11	56	165	F
**19**.	HW	18.9	43rd	14	42	149	F
**20**.	HW	17.8	54th	11	30	130	F
**21**.	HW	16	62nd	4	16	100	M
**22**.	HW	15.3	37th	8	40	140	M

OW = overweight, HW = health weight

The blood biochemical analyses usually considered for obese patients were determined in the same day with saliva samples collection. The results of glycaemia, total plasma cholesterol, HDL-cholesterol and triglycerides are presented as mean ± SD in Table 2.

**Table 2:** The level of blood parameters (total cholesterol, HDL-cholesterol, triglycerides, glycaemia, transaminases) of the children diagnosed with obesity/ overweight and healthy weight included in the study.

		Obese/ Overweight Mean ± SD	Normal Weight Mean ± SD	Student t-Test (vs. NW group)
**Total Cholesterol** **(mg/dl)**		192.5±72.77	124.5±14.32	P = 0.0471
**HDL-Cholesterol** **(mg/dl)**		46.26±18.7	44.56±3.74	P = 0.8407
**Triglycerides** **(mg/dl)**		88.68±35.01	79.7±13.62	P = 0.5384
**Glycaemia** **(mg/dl)**		84.59±10.05	90.86±7.07	P = 0.2294
**Transaminases** **(U/L)**	**AST**	26.26±13.52	24.66±3.13	P = 0.7989
	**ALT**	28.13±23.05	26.64±9.58	P = 0.8908

Normal range of blood parameters: Total Choesterol <170mg/dL, HDL-cholesterol >35 mg/mL, Triglycerides 30-125mg/dL, Glycaemia 70-127mg/mL, Transaminases AST 10-40U/L, ALT 5–30 U/L. NW = normal weight group.

In case of the children selected for the study, total cholesterol was slightly increased in case of obese and overweight children comparing to normal weight group in Table 2. Most of the obese children presented a normal level of plasmatic cholesterol even in cases of high BMI (99^th^ percentile) and no correlation was established between BMI and blood cholesterol level (r = 0.124, P = 0.58). HDL-cholesterol, a parameter better correlated in medical practice with the atherosclerotic risk, was in normal range in case of normal weight group and 15 obese/overweight children, except for two obese children with a slight decreased level comparing to normal range. Triglycerides level and glycaemia are not significantly different (P>0.05) in obese/ overweight group comparing to lean children group (Table 2). Only one child presented an increased level of triglycerides (159 mg%) associated with a normal level of glycaemia.

Normal values of transaminases were noticed in case of obese/ overweight group (Table 2) except for one child with a slightly increased level of transaminases (50-80U/L) associated with a high level of cholesterol (>170mg%) and a decreased level of HDL-cholesterol (<35mg%) in blood.

In case of obese/ overweight group the salivary level of IL-6 was 4.5 times higher in comparison with the normal weight group (Figs 1 and 2) and the results were statistically significant (P<0.05). The level of salivary IL-6 was correlated (r = 0.71, P<0.0002) with BMI and a weak correlated was established with age (r = 0.59, P = 0.07) (Figs 2 and 5).

The IL-6 was elevated in 15 cases, only two overweight children presented salivary concentrations similar to normal weight group. The child with the highest degree of obesity from the group (>99^th^ percentile) presented the highest salivary level of IL-6 (98 ng/mL), followed by two children (96^th^ and 98^th^ percentiles) with a IL-6 level of 61.2ng/mL.

Salivary leptin level was 3 times higher in case of obese/overweight group comparing to control group (p<0.01) (Figs 3 and 4). The salivary leptin level was not correlated nor with BMI (r = 0.1, P = 0.68) or age (r = 0.03, P = 0.07) (Figs 4 and 5). The highest level of leptin was determined in case of an obese child (>99^th^ percentile) diagnosed with asthma (97.5 ng/mL) associated with an increased level of IL-6 (58.6 ng/mL) followed by an overweight child (88^th^ percentile) with 97.4 ng/mL salivary leptin associated with a moderate increased level of IL-6 (23.06 ng/mL). Even both cytokines are secreted by adipose tissue and are involved in inflammation pathways, the salivary level of leptin was not correlated with the IL-6 level (r = 0.07, P = 0.41), six cases of obesity were characterized by elevated IL-6 and normal level of salivary leptin.

### Discussions

In obesity, besides determining the BMI, the physicians require some blood analyses for a better evaluation and monitoring of the associated risks. The most determined biochemical analyses comprise the evaluation of glycaemia, blood lipid profile and transaminases for the investigation of the type 2 diabetes, cardiovascular or hepatic risk [14, 15]. [22,23]. Obesity is also known to be associated with low grade inflammation and oxidative stress which play essential roles in developing further insulin-resistance and metabolic syndrome [2,22–24]. In spite of that, in case of obese patients the presence of systemic inflammation is less considered for determination in the usual medical examination.

The pro-inflammatory signaling molecules generated by adipocytes in obesity like IL-6 and leptin reach high systemic levels and trigger insulin resistance, developing type 2 diabetes and other complications [3,25]. Determination of these markers could be more relevant in a complete obesity diagnostic, revealing an inflammatory status difficult to evaluate by standard biochemical analyses.

Interleukin-6 is a cytokine secreted by adipose tissue being involved in signaling pathways of inflammation and insulin signaling in various tissues and cell types and high circulatory level had been correlated with insulin resistance and type 2 diabetes [25]. Elevated level of IL-6 in blood had been observed in obese patients and several studies demonstrated a strong positive correlation between IL-6 plasma concentration and the salivary level [9,26].

In obese/ overweight group, 15 children had elevated salivary level of IL-6, five of them presenting normal levels of all blood biochemical analyses. One of the highest levels of Il-6 was determined in case of an asthmatic obese patient who presented also the highest level of leptin, sustaining an inflammatory state which could be hardly revealed by the blood analyses. In this particular case, obesity is a known risk factor for asthma, both conditions inducing an elevation of the pro-inflammatory cytokines concentration, especially IL-6 in blood [7,27]. Peters et al., demonstrated that a minority group of obese asthmatics characterized by high levels of IL-6 were more likely to have metabolic dysfunction especially hypertension and systemic leukocytosis [7]. Moreover, IL-6 is considered to have a causal role in severe asthma and sustain the utility of monitoring this cytokine as biomarker in IL-6 high asthma [7,27].

Leptin is an adiposity signaling molecule involved in the control of energy intake and expenditure. High levels of leptin were observed in obese patients sustaining the process of leptin resistance which is responsible of the imbalanced body weight regulation [17,28,29]. Increased levels of salivary leptin were observed in case of 11 children belonging to the obese/overweight group comparing to the normal weight group, 8 of them presenting both pro-inflammatory cytokine increased in saliva. Leptin resistance is variable among the obese patients [28], and may depend on the nutrients consumed from the diet. Some studies sustain the correlation of the high levels of circulatory leptin with diet obesity and an improvement of leptin level was observed after a balanced distribution in the dietary pattern [29–31].

The children included in the obese/ overweight group had no other endocrine disorders or oral cavity infections or inflammation which could interfere the analyses of the saliva samples [32]. All children of this group had at least one pro-inflammatory cytokine increased in saliva and most of them presented blood analyses in normal rage except for some cases of increased total cholesterol and few cases of slightly decreased HDL-cholesterol.

An increased level of the pro-inflammatory cytokines in saliva is revealing a state of systemic inflammation. Even the diagnostic was according to the body mass index and the risk was monitored by usual recommended blood biochemical analyses, these standard diagnostic parameters weren’t enough to predict the presence of inflammation in case of obese/ overweight group.

Because of the health risk associated to weight gain and sustained by the presence of inflammatory status and considering the age of the participants, the medical recommendation for all obese/overweight group was a normal balanced diet and physical exercises without medication.

## Conclusions

The obesity is mainly diagnosed by BMI and several standard blood analyses are recommended to evaluate the potential associated risks such as type 2 diabetes, cardiovascular risk or metabolic syndrome. The low grade inflammatory status present in obesity triggers the associated comorbidities and this is less considered within the medical examination. The results obtained within the study sustain the determination of the pro-inflammatory cytokine, IL-6 and leptin which proved to be efficient in the evaluation of the health status in the obese/ overweight group and could reveal an early inflammatory state, in spite of the normal range of well-known blood biomarkers. Moreover, the level of interleukin-6 was strong correlated with BMI which is the general accepted method used for the assessment of the obesity or overweight degree.

The determination of these markers in saliva samples by a stochastic method proved the utility within the medical examination for a better evaluation of the health status in obesity. The method has some advantages like: easy collection of the biological sample, fast determination of low concentrations and could be promising in case of no associated oral cavity infections or inflammations which could interfere the results. Future studies should consider the fluctuation of these pro-inflammatory markers present in saliva of the obese/overweight patients who are diagnosed with other diseases like endocrine disorders and the evolution of these parameters during the healthy diet program.
